# The most severe HIV epidemic in Europe: Ukraine’s national HIV prevalence estimates for 2007

**DOI:** 10.1136/sti.2008.031195

**Published:** 2008-07-22

**Authors:** Y V Kruglov, Y V Kobyshcha, T Salyuk, O Varetska, A Shakarishvili, V P Saldanha

**Affiliations:** 1 Ukrainian AIDS Centre, Ministry of Health of Ukraine; 2 World Health Organization, Ukraine; 3 International HIV/AIDS Alliance in Ukraine; 4 Joint United Nations Programme on HIV/AIDS, Ukraine

## Abstract

**Objective:**

To revise the national HIV estimates and quantify the magnitude of the HIV epidemic in Ukraine at the end of 2007.

**Design:**

Internationally recommended methods—the Workbook and Spectrum—were employed to generate the estimates. This enables comparison of results with other countries using the same methodology.

**Methods:**

Estimation of the size of most at-risk populations nationally was performed using capture-recapture, multiplier and triangulation methods. HIV prevalence among most at-risk populations was estimated by linked HIV sentinel and behavioural surveillance among injecting drug users, and men who have sex with men, and unlinked sentinel surveillance among sex workers. The range of HIV prevalence and extrapolation for populations at lower risk were determined by consensus among national stakeholders. Results were reviewed by national stakeholders and endorsed by the government of Ukraine.

**Results:**

At the end of 2007, an estimated 395 000 adults (range 230 000–573 000) aged 15–49 were living with HIV in Ukraine. Adult HIV prevalence was estimated at 1.63%, which represents the highest adult HIV prevalence of any country in Europe.

**Conclusions:**

The HIV epidemic in Ukraine continues to grow at a record pace, concentrated among most at-risk populations, the majority of whom are unaware of their HIV status. The results emphasise the need to accelerate the coverage and quality of prevention programmes among most at-risk populations and their sexual partners.

The HIV epidemic in Ukraine remains concentrated among most-at-risk populations (MARPs), including injecting drug users (IDUs), sex workers (SWs), men who have sex with men (MSM) and sexual partners of these populations. Twenty years since its establishment, the national surveillance system for HIV infection continues to be based on HIV case reporting. This surveillance system has been more successful at identifying HIV infection among IDUs than SWs and MSM.^1^ Sentinel surveillance among MARPs has indicated further under-reporting of officially registered cases of HIV infection among these populations, with a number of regions reporting consistently high prevalence rates in some of these groups.

The previous HIV/AIDS estimates in Ukraine (2006),^2^ based on the best data available at the time, indicated 377 600 people living with HIV infection in Ukraine at the end of 2005. These estimates included 344 000 people living with HIV aged 15–49, representing 1.46% of the adult population of Ukraine.

Since 2005 the HIV epidemic in Ukraine has continued to grow. As of the end of 2007, over 122 500 people had been officially registered with HIV infection since the HIV surveillance began in 1987. In 2007, a record number of newly diagnosed individuals with HIV infection were reported—17 699—or an increase of 10% from 2006. Clinical progression from HIV infection to AIDS and death is occurring with greater frequency in Ukraine. At the end of 2007, 22 424 people had been diagnosed with AIDS since the beginning of the epidemic, including 12 490 who had died of AIDS. The number of reported AIDS cases includes 4573 people who were newly diagnosed with AIDS in 2007, a slight decrease of 3.2% from the previous year. AIDS mortality also increased over time, with 2507 AIDS-related deaths reported in 2007.^1^

These officially reported data underestimate the scale of the HIV epidemic in Ukraine as they provide information only about individuals who have been tested, diagnosed with HIV and included into the official national registry. With an indeterminate proportion of the population unaware that they are infected with HIV, it was important to develop up-to-date and accurate estimates of the magnitude of the HIV epidemic in Ukraine. These estimates were also essential to improve access to voluntary counselling and testing (VCT) services for the general population as well as for MARPs.

In 2007 the Ukrainian AIDS Centre under the Ministry of Health decided to revise its national estimates for HIV/AIDS by using the Workbook and Spectrum tools, based on the recommendations of the Joint United Nations Programme on HIV/AIDS (UNAIDS) Reference Group on Estimates, Modelling and Projections.^3^ The 2007 estimates were developed in Kyiv (Kiev) by a national working group led by the Ukrainian AIDS centre, with technical support provided by the World Health Organization and UNAIDS, and in partnership with the International HIV/AIDS Alliance and other organisations. The estimates were subsequently reviewed by national stakeholders, and then endorsed by the Ministry of Health. The estimates also drew on the results of recent research into the size of MARPs and their behaviours. This paper describes the methods and sources of data, as well as the results of these estimates and their significance for Ukraine’s response to the HIV epidemic.

## METHODS

In late 2007 the national working group conducted a series of technical meetings to review the process for generating new estimates and the data needs. In order to be consistent with the methodology used for generating previous estimates for 2005, the national working group agreed to use Workbook and Spectrum.

The working group selected the Workbook Method A as the most appropriate for Ukraine, allowing for estimates to be based on MARPs and their sexual partners. The most recent data among antenatal clinic attendees indicate that HIV prevalence among pregnant women is increasing, from 0.45% in 2005 to 0.52% in 2007, but remains significantly lower than among MARPs. Estimates based on antenatal care data (Workbook Method B) would have risked a significant underestimation of the magnitude of the epidemic, which remains concentrated among MARPs.

The key data requirements for Method A in Workbook were up-to-date and reliable estimates of the size of and HIV prevalence among IDUs, SWs and MSM, and their sexual partners. For each of these MARPs, high and low estimates were made for the population size, based on the most recent research^4^ conducted in 2004–5, which were also used to generate the previous estimates in 2005. The methods for estimating the size of these populations were consistent with international recommendations, based on a combination of capture-recapture, multiplier and triangulation methods.^4^ ^5^ While there might have been minor fluctuations in the size of some of these populations since 2005, it was agreed that the extent of these changes were not believed to undermine the reliability of the existing data.

In many cases, the research also generated disaggregated data for the size of these populations at the regional level. Owing to the limitations of the research design, it was determined that the accuracy of the population estimates for specific regions was limited, and thus only the aggregated national estimates would be used for the development of new national estimates. For the purpose of simplicity and operational ease, the figures were rounded to the closest thousandth.

The following data were used as inputs for 2007 estimates into Workbook on the size of MARPs and the HIV prevalence rates among these groups.

### Estimation of size of populations at higher risk (PHR)

- *Estimated number of IDUs:* from 325 000 to 425 000. In Ukraine, there are also frequent occurrences of injecting drug use among SWs. The estimated numbers of IDUs include SWs who are also engaged in injecting drug use, who otherwise were not included in the estimated number of SWs, as injecting drug use was considered to be a higher behavioural risk for HIV. Thus, overlap between such populations in these estimates was avoided.
- *Estimated number of SWs:* from 110 000 to 250 000. This estimate includes only female SWs, as the group of male SWs is believed to be smaller and harder to reach than female SWs. Only street-based SWs who could be reached by current prevention programmes and HIV surveillance studies were included in the estimates.
- *Estimated number of MSM:* from 177 000 to 430 000. The low population estimate was generated by triangulation research, whereas the high estimate was based on the internationally recognised conservative estimate of the percentage of the male population that practises sex with men—3% of the adult male population.
- *Estimated number of clients of SWs:* from 330 000 to 750 000. No specialised research has been conducted in Ukraine to estimate the size of this specific population, so it was agreed to use the average number of clients reported by SWs per week, which ranged from two to six.^6^ The average of three clients per SW, per week, was used to calculate the number and range of male clients. There remain concerns that these figures may underestimate the number of clients of SWs, but no other data are currently available.
- *Number of prisoners:* 130 000. It was also agreed to add prisoners as an additional MARP, as the size of this population and data on HIV prevalence are available and of high quality. The addition of prisoners was not meant to indicate that imprisonment is considered to be a risk factor for HIV infection. Instead, it is suspected that there is a high prevalence of injecting drug use and a high proportion of MSM among prisoners. The sizes of the groups of IDUs or MSM who may be in prison were included in the calculations for prisoners, as they were not included in the estimates of other MARPs.

### Estimation of size of populations at lower risk (PLR)

In addition to the size of MARPs, the Workbook methodology also requires estimates on the size of so-called “bridge populations” that are at lower risk for HIV. Specific research has not been conducted in Ukraine to develop reliable estimates of the size and HIV prevalence for these groups. It was agreed that the size estimates for these groups would be based on relevant data drawn from recent behavioural studies among MARPs and informed by the opinions of national and international experts, with all assumptions about HIV prevalence being made explicit for each of the bridge populations.^6^^–^9

- *Estimated number of sexual partners of IDUs:* The number of sexual partners of IDUs was calculated on the basis of results of a special survey on IDU behaviour which indicated that an IDU had on average between one and three sexual partners.^7^ The coefficient of 1.3 sexual partners per IDU was applied to estimate the number of sexual partners of IDUs. The final range used for the national estimations was from 422 500 to 552 500 sexual partners of IDUs.
- *Estimated number of female partners of MSM:* The number of female partners of MSM was calculated on the basis of results of a special survey on MSM behaviour,^8^ which indicated that 33% of MSM had an average of three female sexual partners. The coefficient of one female sexual partner per MSM was applied to an estimated number of female sexual partners of MSM. The final range used for the national estimations was from 177 000 to 430 000 female partners of MSM.
- *Estimated number of sexual partners of clients of SWs:* The number of sexual partners of clients of SWs was calculated on the basis of the preliminary research into behaviours among bridge populations (International HIV/AIDS Alliance in Ukraine, Report on Bridge Populations, Kyiv, 2006, unpublished), which indicated that each client had an average of an additional 2.5 non-commercial sexual partners. The coefficient of 2.5 sexual partners per SW client was applied to the estimated number of sexual partners of SW clients. The final range used was from 825 000 to 1 875 000 sexual partners of clients of SWs. It was assumed that all of the sexual partners of the clients of female SWs were female.

### HIV prevalence data for 2007 estimates

In 2007, the Ukrainian AIDS Centre conducted a linked HIV biological and behavioural surveillance survey in different cities of Ukraine to determine the HIV prevalence among IDUs in six sites and MSM in four sites using respondent driven sampling (RDS).^1^ The HIV prevalence ranged from 10.4% (city of Lugansk) to 87.8% (city of Krivoy Rog) among IDUs, and from 4.4% (city of Kyiv) to 23.2% (city of Odessa) among MSM. Subsequent analysis of the HIV prevalence data for IDUs with RDS tools provided a range that eliminated the outlier values. For the IDU data entry into Workbook, it was agreed to use the prevalence range of 17.3% to 70%. Given the small number of sentinel sites for MSM, it was agreed not to eliminate the outlier values and for the data entry into Workbook to use rounded prevalence values of 4% to 23%.

The most recent data on HIV prevalence among non-IDU SWs was collected in 2007, through unlinked biological sentinel surveillance in nine sites, with a range of HIV prevalence from 4% (city of Kyiv) to 31% (city of Poltava). It was agreed to eliminate the outlier values and to use the prevalence range of 8% to 30% for the data entry into Workbook.

For prisoners, the HIV prevalence data were based on the latest results of HIV surveillance from the state prison department, with the low prevalence taken from the lower range of the results of regular surveillance among prisoners, and the high prevalence taken from the upper range of results from sentinel surveillance in selected prisons (unpublished data from the state prison department, 2007).

For the population of male clients of FSWs and the other populations at lower risk, there is no sentinel surveillance data available in Ukraine. Therefore, the working group used best estimates of the likely HIV prevalence rates, with the range being linked, but consistently lower than the range of HIV prevalence among the populations at higher risk.

As Ukraine is a country with a concentrated HIV epidemic among specific MARPs, existing estimates of the size of populations at higher and lower risk, as well as data on HIV prevalence in these groups, were entered into the Workbook spreadsheet model^9^ to develop the point prevalence among adults in Ukraine as of the end of 2007. The final sets of inputs for Workbook are presented in table 1.

**Table 1:** Estimated population size and HIV prevalence rates among populations at higher and lower risk, Ukraine, end of 2007

Population groups	Population size estimate		HIV prevalence estimate	
	Low estimate	High estimate	Low	High
Populations at higher risk:				
IDUs	325 000	425 000	17.30%	70.00%
MSM	177 000	430 000	4.00%	23.00%
SWs (female)	110 000	250 000	8.00%	30.00%
Male clients of SWs	330 000	750 000	2.00%	5.00%
Prisoners	130 000	130 000	3.50%	12.0%
Populations at lower risk that are not included in PHR:				
Partners of IDU	422 500	552 500	8.00%	30.00%
Female partners of MSM	177 000	430 000	1.30%	7.60%
Partners of clients of SWs	825 000	1 875 000	1.16%	2.00%

IDUs, injecting drug users; MSM, men who have sex with men; SWs, sex workers; PHR, populations at higher risk.

**Figure 1:**
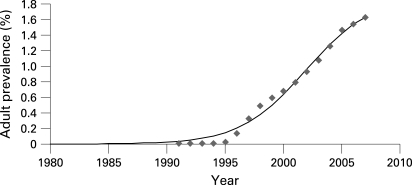
HIV point prevalence curve in Ukraine, 1987–2007.

The HIV point prevalences for 2003 and 2005 were based on the results of the previous national HIV estimates generated by Workbook, 1.08% and 1.46%, respectively. However, the data required to generate estimates for previous years were not available. In order to develop data for the missing HIV point prevalence rates, it was assumed that in previous years, the national HIV-prevalence rate was 1.5 times higher than the officially reported number of registered cases of HIV. This coefficient of 1.5 was used to multiply the number of officially registered cases for the earlier years of the epidemic. For interval years where no estimates were generated, the HIV point prevalence was calculated as the average between the two existing prevalence points, thus providing a complete point prevalence curve for each year since 1991 (fig 1).

The Workbook outputs with the point prevalence were entered into the Spectrum Policy Modelling System (Spectrum).^10^ Spectrum was used to generate detailed estimates for HIV and AIDS for Ukraine as of the end of 2007, and projections for the period up to 2013. Inputs were made to the “Epidemiology” section under the AIDS (AIM) module in Spectrum. The HIV prevalence curve was imported from the Workbook file into Spectrum with the following values (table 2).

**Table 2:** Point prevalence of HIV infection by years, Ukraine, 1990–2007

Year	HIV point prevalence
1990	0.00%
1991	0.01%
1992	0.01%
1993	0.01%
1994	0.01%
1995	0.03%
1996	0.14%
1997	0.33%
1998	0.49%
1999	0.59%
2000	0.68%
2001	0.79%
2002	0.93%
2003	1.08%
2004	1.26%
2005	1.46%
2007	1.63%

The following assumptions were made for the other Spectrum inputs:

- The percentage of all pregnant women receiving prevention of mother-to-child (PMTCT) services was 86% in 2003, with a gradual increase to 93% over the period of 2004–10. The rate of mother-to-child transmission in the absence of the PMTCT programme was 32% (before 2003) and 10%, which is based on a combination of zidovudine (AZT) and single-dose nevirapine.
- The percentage of those on antiretroviral treatment (ART) surviving to the following year was assumed to be 85%.
- The “regular progression” model of HIV progression was used as the variable for the speed of HIV progression.

Using Spectrum, these same data were used to determine the range of these estimates. Using the uncertainty analysis function, Spectrum generated the ranges for the adult HIV prevalence based on plausibility bounds of 95%.

The outputs from Workbook and Spectrum were reviewed and revised during a series of meetings by the working group before they were finalised in January 2008. The final step in the process was the review of the new estimates at a public meeting of the stakeholders, chaired by the Minister of Health of Ukraine. The final estimates were subsequently reflected in Ukraine’s 2008 Report on Monitoring Progress towards the UNGASS Declaration of Commitment on HIV/AIDS.

## RESULTS

The results from Workbook indicate that at the end of 2007, there were an estimated 395 296 adults (aged 15–49) living with HIV in Ukraine (table 3). The adult HIV prevalence was estimated at 1.63%, which represents the highest adult HIV prevalence of any country in Europe. Based on the uncertainty analysis generated by Spectrum, the range for the adult HIV prevalence was between 0.95% and 2.37%—equal to 230 000–573 000 adults (15–49) living with HIV.

**Table 3:** Estimated number of people living with HIV by groups of the populations at higher and lower risk, and estimated adult HIV prevalence, Ukraine, end of 2007

Population group	Number
IDUs		164 000
MSM		41 000
FSWs		34 000
Male clients of FSWs		19 000
Prisoners		10 000
**Subtotal PHR**		**268 000**
Partners of IDU		93 000
Female partners of MSM		13 500
Partners of clients of FSWs		21 000
**Subtotal PLR**		**127 000**
**Total number of adults (15–49) living with HIV**		**395 000**
Number of women (15–49) living with HIV		178 000
Percentage of people living with HIV who are female		45.0%
Percentage of total people living with HIV that are IDU		41.4%
**Percentage of adult (aged 15–49) prevalence**		**1.63%**

IDUs, injecting drug users; MSM, men who have sex with men; FSWs, female sex workers; PHR, populations at higher risk; PLR, populations at lower risk.

The results from Workbook indicate that 163 688 IDUs are estimated to be living with HIV, representing 41.4% of all adults living with HIV. This indicates that between 38.5% and 50.3% of the estimated population of IDUs are already infected with HIV. The proportion of IDUs among adults living with HIV is closely correlated with the official surveillance data which estimates 40% of newly reported cases of HIV in 2007 being attributed to IDUs.

Among SWs, the estimated HIV prevalence is high, ranging from 13.6% to 31.0% of the estimated SW population. The data also indicate that 10.0% to 23.0% of the estimated population of MSM are currently living with HIV.

These results indicate that the gap is narrowing between the number of adult men and women with HIV, with women representing 45% of the adults living with HIV. These data are largely consistent with the officially reported ratio between men and women, with women accounting for 43.8% of all the newly registered cases of HIV in 2007.

Spectrum also enabled us to expand the estimates for the entire population, indicating the overall HIV prevalence of 1.08%. These data from Spectrum indicate an additional 44 585 people living with HIV outside the age range of 15–49 years. Thus, a total of 439 881 people were estimated to be living with HIV in Ukraine, including 251 330 males and 188 551 females. However, the working group agreed to use the prevalence figure of 1.63% as the official estimates for 2007, as this figure is based on the estimated adult prevalence for the age group 15–49, and thus can be compared to other countries using the same methodology.

## DISCUSSION

The most sobering conclusion that can be drawn from the 2007 estimates is that the HIV epidemic in Ukraine continues to grow at a record pace. With a high and growing adult HIV prevalence, Ukraine maintains its unfortunate distinction of having the highest HIV prevalence of any country in Europe. In comparison to the previous 2005 estimates, the 2007 estimates indicate the epidemic continues to spread, mainly among MARPs, many of whom remain unaware of their HIV status. This has serious implications for prevention programmes among these groups. These programmes need to focus more on VCT and behaviour change, as well as providing access to care, treatment and support services.

The 2007 estimates reflect the opinions among an extensive range of national and international experts who participated in the process of generating these estimates. These estimates are considered valid and currently represent the most accurate and comprehensive HIV estimates available for Ukraine at the end of 2007. Key information, particularly information on size of the populations such as sexual partners of the MARPs and HIV prevalence data among these groups, is still lacking. For these reasons, these estimates should be cited and used with a degree of caution until more relevant data become available. In particular, the use of the Workbook method is highly sensitive to the results of sentinel surveillance data from MARPs, which are extremely high in Ukraine. The authors acknowledge that the transposition of the sentinel surveillance data may have resulted in some degree of overestimation of national HIV prevalence. In order to minimise such risks, the authors propose to pilot the use of the Estimations and Projection Package (EPP) to generate and compare future national HIV estimates.

The process of developing these estimates underscores the importance of generating a national consensus on the status of the HIV epidemic. As a result of extensive consultations and the strategic dissemination of the results, national stakeholders, including high-level decision-makers such as the President of Ukraine and the Minister of Health, have accurately cited these estimates. The estimates depict the magnitude of the epidemic, and provide a robust epidemiological baseline for the development of a new national AIDS programme in Ukraine for the next five years.
